# Physical Appearance Perfectionism: Psychometric Properties and Factor Structure of an Assessment Instrument in a Representative Sample of Males

**DOI:** 10.3389/fpsyg.2022.806460

**Published:** 2022-02-18

**Authors:** Robin Rica, María Solar, Alba Moreno-Encinas, Sara Foguet, Emilio Juan Compte, Ana Rosa Sepúlveda

**Affiliations:** ^1^Faculty of Psychology, Autonomous University of Madrid, Madrid, Spain; ^2^Escuela de Psicología, Universidad Adolfo Ibáñez, Peñalolén, Chile

**Keywords:** PAPS-Spanish version, body image, male body image, body dissatisfaction measure, male students

## Abstract

Perfectionism is a multidimensional construct with both positive and negative aspects. Recently, the concept of appearance-oriented perfectionism has been introduced, which is associated with body image dissatisfaction and weight and shape control behaviors. The Physical Appearance Perfectionism Scale (PAPS) is a 12-item two-factor instrument developed to assess this new dimension of perfectionism. The aim of the study is to validate the Spanish version of PAPS among a representative sample of 850 male university students in Spain (*M* = 20 years old; *SD* = 2.7). Exploratory and confirmatory factorial structure, internal consistency, convergent and concurrent validity, and associated predictor variables analyses have been carried out. Results showed that the Spanish version of the PAPS maintains the original factor structure with all items and proves to be a reliable instrument. Physical appearance-oriented perfectionism is associated with general perfectionism, higher body dissatisfaction, Eating Disorders and Muscle Dysmorphia symptomatology, and compulsive exercise, particularly in the Worry about Imperfection subscale. These variables also act as predictors of physical appearance perfectionism. The use of the PAPS-S and the analysis of its subscales is recommended in the context of body image-related pathologies such as Eating Disorders and Muscle Dysmorphia.

## Introduction

Perfectionism is a multidimensional and multifaceted personality disposition characterized by striving for flawlessness and setting exceedingly high standards of performance accompanied by overly critical evaluations of one’s behavior (Frost et al., 1990; Hewitt and Flett, 1991; Stoeber et al., 2015). Given the great importance they attach to external evaluation, perfectionists feel pressure to perform at their best to avoid disapproval or disappointment from others (Frost et al., 1990; Hewitt and Flett, 1991).

Frost et al. (1993) found that perfectionism was distributed into two dimensions that captured both negative and positive aspects: maladaptive evaluative concerns and positive achievement striving. This distinction is important, as only the components of the first dimension are related to psychopathology, such as negative affect, depression, anxiety, rumination and avoidance coping, emotional dysregulation, body image dissatisfaction (i.e., both muscularity and thinness oriented), compulsive exercise, obsessive-compulsive disorders, eating disorders (ED) or muscle dysmorphia (MD) (Frost et al., 1993; Grammas and Schwartz, 2009; Murray et al., 2012; Donahue et al., 2018; Bergunde and Dritschel, 2020; Çakin et al., 2021).

Recently, Yang and Stoeber (2012) introduced the concept of physical appearance perfectionism, which is composed of two components: Hope for Perfection (HFP) and Worry about Imperfection (WAI). The first one relates to approach-oriented perfectionistic strivings, associated with the positive reinforcement that comes from achieving attractiveness or admiration. The second component is related to aspects or avoidance-oriented perfectionistic concerns, linked to attempts to avoid imperfection, disapproval and criticism (Stoeber and Yang, 2015). Appearance-oriented perfectionism, as a specific domain, is also positively associated, without sex differences, with social anxiety related to appearance, appearance disturbance, body shape disturbance, body image concerns, body weight control behaviors; and is negatively associated with body appearance self-esteem (Yang and Stoeber, 2012; Simon et al., 2022).

To evaluate the desire for a perfect physical appearance, the Physical Appearance Perfectionism Scale (PAPS; Yang and Stoeber, 2012) was developed. The PAPS is a 12-item instrument with five alternative Likert-type responses from 1 (i.e., totally disagree) to 5 (i.e., totally agree) that presents a two-factor structure, differentiating between maladaptive concerns (i.e., WAI, seven items) and positive strivings (i.e., HFP, five items), which are all aspects of physical appearance perfectionism. “*I am never happy with my appearance no matter how I dress*” is an example from the WAI subscale, while *“I hope my body shape is perfect*” is an item from de HFP subscale. The original study validated the scale in a mixed sample of students in both China (47.4% male) and United Kingdom (20.5% male), with high reliability rates for both the Chinese and the English samples. The PAPS has also been used in another Chinese adolescent sample showing good indices of internal consistency and fit to the original factor structure (Yang et al., 2017). A recent study using the PAPS on a sample of female university students in the United Kingdom, slightly modifying the Hope for Perfection subscale (i.e., “*hope*” was replaced with “*strive*”), also found good reliability indices and replicated the two-factor structure (Bergunde and Dritschel, 2020). The PAPS has recently been adapted and validated to Brazilian Portuguese in a mixed sample of adults (i.e., 49.4% males) providing satisfactory indices of internal consistency and maintaining the two-factor structure by removing items 1 and 2 (Ferreira et al., 2018; Neves et al., 2019).

English, Chinese and Brazilian Portuguese adaptations make the PAPS a widely applicable instrument. However, so far, no adaptation and validation has been made to Spanish. On the other hand, none of the studies using the PAPS found sex differences in its factor structure (Yang and Stoeber, 2012; Yang et al., 2017; Neves et al., 2019; Bergunde and Dritschel, 2020), which is consistent with previous studies on general aspects of perfectionism (Frost et al., 1993). However, although the relationship of PAPS with ED symptomatology has been studied, it has not been explored in the context of male body dissatisfaction related to muscularity-oriented and MD symptomatology, which is more prevalent in males (Pope et al., 2000).

The main objective of this study was to fill this research gap and explore the factorial structure of the Spanish translation of the PAPS in a representative sample of Spanish university men. The aims were: (a) to assess the factor structure of the instrument, (b) its reliability, (c) its convergent validity with general perfectionism, (d) its concurrent validity with body dissatisfaction, ED and MD symptomatology, and compulsive exercise, and (e) and to explore the associated predictor variables. We hypothesized that: (1) the original two-factor structure would be supported, (2) the test would show good reliability, (3) students with greater levels of physical appearance perfectionism would be associated positively with general perfectionism and a greater body dissatisfaction, ED and MD symptomatology. Specifically, we expect higher levels of Worry about imperfection to be more strongly associated with psychopathological variables than scores on the Hope for Perfection subscale. For the latter, we expect stronger associations with the apparently positive aspects of perfectionism (i.e., achievement expectations and organization). Finally (4), the study variables are expected to act as predictor variables associated with physical appearance perfectionism.

## Materials and Methods

### Participants

Among the 21 schools on the university campus, the five schools with the highest number of male students enrolled (i.e., over 70%) were selected. In this manner, a total of 1634 students were targeted. To achieve a representative sample of the university campus by school, the sample design was proportionally stratified according to this variable, assuming a 95% confidence interval and 0.05 of sampling error. A total of 1088 students were identified as the desired sample size.

The final sample comprised 850 male university students from different degrees: (1) Physical Activity and Sports Sciences from Polytechnic (*n* = 297; 91.1% response rate), (2) Physics (*n* = 92; 96.8 response rate), (3) Economics (*n* = 171; 77.7% response rate), (4) Computer Science Engineering (*n* = 114; 49.6% response rate), and (5) Business Administration and Management (*n* = 176; 81.1% response rate). The mean age of the sample was 20 years old (*SD* = 2.7). The mean Body Mass Index (BMI) was 22.4 (*SD* = 2.8).

### Measures

In addition to the PAPS, students answered a set of questions regarding their age, and nationality. Participants also reported their height and their weight, allowing us to calculate an estimate of their BMI (kg/m^2^). The participants also completed the following measures:

**Multidimensional Perfectionism Scale** (MPS; Frost et al., 1990; Carrasco et al., 2009): 35-items questionnaire with 5 Likert answer options from 1 (*strongly disagree*) to 5 (*strongly agree*). In its original version, the MPS provides a total score and 6 subscales (i.e., Concern over Mistakes, Personal Standards, Parental Expectations, Parental Criticism, Doubt about Actions and Organization). The Spanish adaptation showed a four-factor structure. The Fear of Mistakes subscale refers to the more negative aspects of perfectionism related to the concern about mistakes and doubts about one’s own actions, the Parental Influences subscale relates to the influence of family expectations and criticism in the genesis of perfectionism, the Achievement Expectations subscale refers to competitiveness and comparison with the performance of others when evaluating one’s own performance, and finally, the Organization subscale refers to the importance of order and organization. The Spanish version has excellent levels of internal consistency (range: α = 0.87 to 0.93). In the current sample, the scale showed an omega score of 0.94.

**Eating Disorder Examination Questionnaire** (EDE-Q; Fairburn and Beglin, 1994; Peláez-Fernández et al., 2012; Rica et al., 2021): The questionnaire has 28 items, that asks directly about attitudes related to key features of ED psychopathology in a 28-day time frame. The same four subscales (i.e., Restraint, Eating concern, Weight concern and Shape concern), of the EDE interview are calculated through 22 attitudinal items, and responses are given on a 7-point Likert-type scale from 0 (*never*) to 6 (*every day*). The EDE-Q global score is obtained by averaging subscales’ scores. The initial Spanish version shows adequate levels of internal consistency (range: α = 0.74 to 0.91) as well a recent validation study in a representative sample of Spanish males (range: omega = 0.72 to 0.93). In the present study, we used the most recent Spanish male sample validation of the EDE-Q, which yields a two-factor structure (i.e., Restraint and Eating, Weight and Shape concern). The first subscale refers to altered eating behaviors to lose or avoid weight gain (e.g., decreasing amounts of food), while the second subscale refers to the presence of ruminations about calorie content, body shape or weight number. In the current sample, the EDE-Q also showed good reliability indices (range: omega = 0.74 to 0.92).

**Male Body Attitudes Scale** (MBAS; Tylka et al., 2005; Sepúlveda et al., 2016): The MBAS measures body dissatisfaction in men and consists of 24 items on a Likert-type scale, with scores between 1 (*never*) and 6 (*always*). In the Spanish version the only two items of the Height subscale were excluded. The internal consistency levels for the total score of the MBAS-S were good, as well as for the subscales of Muscularity and Low Body Fat (range: α = 0.85 to 0.88). The Muscularity subscale relates to the presence of concern about muscle bulk and the pursuit of greater muscle development, while the Low Body Fat subscale refers to concern derived from the rejection of body fat that hinders muscle visibility and the feeling of being fat. In the current sample, MBAS showed good reliability indices (range: omega = 0.84 to 0.94).

**Muscle Dysmorphic Disorder Inventory** (MDDI; Hildebrandt et al., 2004; Sepúlveda et al., 2019): Questionnaire of 13 items with a response range from 1 (*never*) to 5 (*always*) that evaluates body dissatisfaction from a male perspective related to muscle development. The MDDI is divided into three subscales and a total score. The Drive for Size subscale refers to the perception of not being sufficiently, the Appearance Intolerance subscale evaluates the presence of avoidance behaviors of displaying one’s own body (e.g., wearing loose clothing) and, finally, the Functional Impairment subscale contains items related to maintaining a routine of excessive exercise, the discomfort of altering this behavior, and the avoidance of social situations. muscular, looking small and the desire to increase body size. The Spanish version showed adequate reliability indices (range: α = 0.73 to 0.85). In the current sample, MDDI showed adequate levels of internal consistency (range: omega = 0.84 to 0.90).

**Compulsive Exercise Test** (CET; Taranis et al., 2011; Author et al., 2022). This is a 24-item self-report questionnaire that uses a 6-point Likert scale, ranging from 0 (*never true*) to 5 (*always true*), with higher scores indicating greater levels of compulsive exercise. In the original validation five factors were identified (i.e., Avoidance and rule-driven behavior, Weight control exercise, Mood improvement, Lack of exercise enjoyment, Exercise rigidity) and total score. The Spanish version shows a brief three-factor structure and 15-items with good reliability indices (range: omega = 0.82 to 0.91). The Avoidance of negative affect factor is related to the avoidance of negative feelings that are experienced when exercise is missed, the Weight control exercise factor refers to exercising to improve appearance or for weight and shape reasons and, the Mood improvement factor is related to the positive mood improvements associated with exercise. In the current sample, the scale showed an Omega score of 0.93.

### Procedure

Permission was requested from the original authors of the PAPS for the cultural adaptation of the instrument into Spanish (Annex 1). A back-translation procedure was then conducted (Brislin, 1970). First, two psychologists translated the English 12-item instrument into Spanish. Second, Spanish items were independently back translated into English by another bilingual psychologist. A small proportion of students (*n* = 10) read the items to ensure that clarity and relevance was expressed for all the items. Finally, the original version was compared with the translation, keeping the items identical, and discussing possible discrepancies.

The tests were administered collectively in the classroom and completed individually in electronic or paper forms after obtaining informed consent, highlighting voluntary participation, confidentiality and anonymity of the responses. The battery could be completed in 30 min. Permission to conduct the study was granted by the university’s deans and the participants’ teachers. Approval was obtained by the Ethics Committee of the University (“MASKED FOR REVIEW,” CEI-75-1368). All procedures performed in this study involving human participants were in accordance with the ethical standards and with de Helsinki Declaration and its later amendments or comparable ethical standards.

### Statistical Analysis

Statistical analyses were carried out using SPSS 25.0, Mplus 7.11, and RStudio, employing the MNV package (Korkmaz et al., 2014) and the psych package (Revelle, 2020). Descriptive statistics (mean ± standard deviation) were calculated for all scale scores. In order to assess the internal structure of the PAPS, a cross-validation was carried out, dividing the total sample into two equivalent random subsamples (Swami and Barron, 2019). There were no significant differences between both subsamples in terms of mean age (*t*_848_ = –1.04; *p* = 0.30) or mean IMC (*t*_848_ = 0.71; *p* = 0.48), as well as degree (χ^2^_4_ = 3.17; *p* = 0.53) or year (χ^2^_2_ = 2.48; *p* = 0.29). One subsample (*n* = 435) was used to test the factor structure proposed by Yang and Stoeber (2012) through a confirmatory factor analysis (CFA) and an Exploratory Structural Equation Modeling (ESEM) approach (Asparouhov and Muthén, 2009) with target rotation. The other subsample (*n* = 415) was used to carry out an Exploratory Factor Analysis (EFA) with oblimin rotation; the number of factors was determined through parallel analysis (Horn, 1965) with an Unweighted Least Squares (ULS) estimator. Mardia’s test revealed that the PAPS did not follow a multivariate normal distribution (skewness = 2426.90, *p* < 0.001; kurtosis = 26.73, *p* < 0.001). Since data were ordinal and non-normal, both analyses were carried out using Robust Weighted Least Squares (WLSMV). Several fit indices were considered in CFA and ESEM analyses: The Root Mean Square Error of Approximation (RMSEA) and its 90% confidence interval, the Tucker Lewis index (TLI), the Comparative Fit Index (CFI), and the Weighted Root Mean Square Residual (WRMR). A model is considered to present a good fit when RMSEA ≤ 0.08 (Browne and Cudeck, 1993; Swami and Barron, 2019), WRMR ≤ 1.0 (DiStefano et al., 2018), and CFI and TLI ≥ 0.95 (Hu and Bentler, 1999; Swami and Barron, 2019). Given the Likert-type nature of the PAPS, internal consistency was assessed using an omega coefficient (McDonald, 1999; Swami and Barron, 2019); values ≥ 0.80 were considered adequate (Nunnally, 1976). In addition, its convergent and concurrent validity was assessed through Spearman correlations with the MPS, EDE-Q, MBAS-S, MDDI and CET-S. Finally, the capacity for the variables measured by the aforementioned tests and for the BMI to predict physical appearance perfectionism was analyzed using multiple hierarchical regression, after checking the assumptions of this kind of analysis.

## Results

### Internal Structure

First, a CFA was carried out in one of the subsamples (*n* = 435). Fit statistics for this analysis are presented in Table 1. The model proposed by Yang and Stoeber (2012) showed a poor fit to our data since fit indices were far from the recommended cut-off points. An examination of modification indices suggested a cross-loading for item 5 (“I worry that my appearance is not good enough”) onto the Hope for Perfection factor (MI = 195.66). Thus, even after excluding item 5 from the analysis, this model continued to show a poor fit. Next, we tested the same model using the ESEM approach. This model showed a good fit, except for RMSEA, with a value above the recommended cut-off point (see Table 1). As expected, item 5 had a significant loading onto the Worry About Imperfection factor (loading = 0.596), but it also had a significant cross-loading onto the Hope for Perfection factor (loading = 0.395). Although there were more significant zero-target cross-loadings, their values were below 0.30 (i.e., between 0 and 0.20).

**TABLE 1:** Fit index values for the tested models.

Models	χ^2^ (d.f.)	RMSEA [C.I.; 90%]	CFI	TLI	WRMR
CFA two-factor model	565.40 (53)	0.150 [0.139 –0.162]	0.909	0.887	1.978
CFA two-factor excluding item 5	333.10 (43)	0.126 [0.113 –0.138]	0.945	0.929	1.607
ESEM two-factor model	239.04 (43)	0.103 [0.091 –0.116]	0.965	0.947	0.858

*All models were tested using one half of the total sample (n = 435).*

We then carried out an EFA analysis using the other subsample (*n* = 415). Parallel analysis results suggested the extraction of two factors. EFA results supported ESEM results, and all items loaded onto both factors in accordance with theoretical expectations (Yang and Stoeber, 2012), although item 5 showed a significant large cross-loading. Factor loadings are presented in Table 2.

**TABLE 2:** Pattern matrix of PAPS items.

	Factor 1	Factor 2
Item 1	**0.753**	–0.044
Item 3	**0.827**	–0.086
Item 5	**0.518**	**0.424**
Item 8	**0.895**	–0.057
Item 9	**0.859**	–0.076
Item 10	**0.617**	0.252
Item 11	**0.763**	0.183
Item 2	0.114	**0.625**
Item 4	–0.024	**0.883**
Item 6	0.019	**0.811**
Item 7	–0.045	**0.891**
Item 12	0.001	**0.889**

*This analysis was carried out using one half of the total sample (n = 415). Loadings with values ≥ 0.30 are bolded.*

### Descriptive Statistics, Internal Consistency, Convergent and Concurrent Validity

Means, standard deviations, and internal consistency for all measures, as well as correlations among all scale scores, are presented in Table 3. The PAPS and its subscales showed excellent internal consistency, with omega coefficient values above 0.90.

**TABLE 3:** Means (standard deviations), internal consistency in the Spanish male sample, and correlations among variables with the PAPS-S.

	Mean (*SD*)	1	2	3	4	5	6	7	8	9	10	11	12	13	14	15	16	17	18	19
1. PAPS-S-WAI	13.92 (5.74)	**0.94**	0.40**	0.87**	0.52**	0.32**	0.28**	0.07*	0.43**	0.25**	0.67**	0.64**	0.47**	0.62**	0.72**	0.45**	0.71**	0.17**	0.60**	0.34**
2. PAPS-S-HFP	15.81 (4.84)		**0.94**	0.80**	0.28**	0.19**	0.35**	0.17**	0.34**	0.21**	0.30**	0.32**	0.30**	0.20**	0.31**	0.35**	0.21**	0.31**	0.42**	0.39**
3. PAPS-S total	29.73 (8.86)			**0.94**	0.49**	0.31**	0.37**	0.14**	0.47**	0.28**	0.60**	0.58**	0.47**	0.51**	0.64**	0.49**	0.58**	0.28**	0.62**	0.43**
4. MPS-FM	24.40 (7.50)				**0.85**	0.58**	0.53**	0.19**	0.83**	0.14**	0.38**	0.35**	0.28**	0.32**	0.39**	0.32**	0.38**	0.26**	0.44**	0.31**
5. MPS-PI	18.09 (6.84)					**0.86**	0.38**	0.10**	0.74**	0.08*	0.27**	0.25**	0.14**	0.22**	0.24**	0.20**	0.25**	0.17**	0.29**	0.26**
6. MPS-AE	26.55 (7.27)						**0.86**	0.41**	0.81**	0.22**	0.21**	0.24**	0.21**	0.15**	0.23**	0.24**	0.19**	0.29**	0.34**	0.32**
7. MPS-O	20.36 (4.91)							**0.89**	0.51**	0.15**	0.10**	0.13**	0.05	0.06	0.05	0.08*	0.07	0.13**	0.13**	0.16**
8. MPS total	89.39 (19.65)								**0.94**	0.20**	0.34**	0.34**	0.25**	0.27**	0.33**	0.30**	0.32**	0.30**	0.43**	0.37**
9. EDE-Q: R	0.89 (1.1)									**0.74**	0.51**	0.71**	0.16**	0.50**	0.38**	0.12**	0.34**	0.32**	0.34**	0.45**
10. EDE-Q: EWSC	0.79 (0.88)										**0.91**	0.97**	0.32**	0.79**	0.69**	0.29**	0.77**	0.21**	0.55**	0.42**
11. EDE-Q total	0.81 (0.84)											**0.92**	0.30**	0.80**	0.68**	0.28**	0.73**	0.27**	0.55**	0.48**
12. MBAS-S-M	2.59 (0.97)												**0.88**	0.24**	0.82**	0.79**	0.27**	0.20**	0.65**	0.24**
13. MBAS-S-LBF	2.07 (0.87)													**0.84**	0.73**	0.17**	0.74**	0.14**	0.43**	0.41**
14. MBAS-S total	2.32 (0.73)														**0.94**	0.65**	0.65**	0.20**	0.70**	0.39**
15. MDDI-DFS	9.77 (4.12)															**0.88**	0.28**	0.29**	0.81**	0.27**
16. MDDI-AI	5.74 (2.63)																**0.84**	0.12**	0.58**	0.27**
17. MDDI-FI	6.58 (3.47)																	**0.86**	0.69**	0.59**
18. MDDI total	4.25 (5.24)																		**0.90**	0.53**
19. CET-S total	2.25 (0.96)																			**0.93**

Omega coefficients are presented along the diagonal in bold. PAPS-S, Physical Appearance Perfectionism Scale, Spanish version; WAI, Worry About Imperfection; HFP, Hope for Perfection; MPS, Multidimensional Perfectionism Scale; FM, Fear of Mistakes; PI, Parental Influence; AE, Achievement Expectations; O, Organization; EDE-Q, Eating Disorders Examination-Questionnaire; R, Restraint; EWSC, Eating, Weight, and Shape Concern; MBAS-S, Male Body Attitudes Scale, Spanish version; M, Muscularity; LBF, Low Body Fat; MDDI, Muscle Dysmorphia Disorder Inventory; DFS, Drive for Size; AI, Appearance Intolerance; FI, Functional Impairment; CET-S, Compulsive Exercise Test, Spanish version. *p < 0.05, **p < 0.01.

Regarding the convergent validity, most correlations between the PAPS-S and the MPS total scores were positive, significant, with mild to high correlations. It is worth mentioning that the correlation between the two total scores, as well as the correlation of Worry About Imperfection and the PAPS-S total score with Fear of Mistakes, which were the highest correlations between both questionnaires.

On the other hand, regarding the concurrent validity, the PAPS-S scores showed moderate to high significant correlations with the EDE-Q total scores and the Eating, Weight and Shape Concern factor. The Worry About Imperfection factor showed the highest correlations with both EDE-Q scores, while the Hope for Perfection factor showed the lowest correlations. However, the correlations between the PAPS-S scores and the Restraint factor were rather low.

The PAPS-S also showed positive moderate to high significant correlations with the MBAS-S scores. As was the case for the EDE-Q, the Worry About Imperfection factor showed the highest correlations with the MBAS-S, while the Hope for Perfection factor showed the lowest correlations. It is worth mentioning that the Muscularity subscale correlations with the PAPS-S were lower than Low Body Fat and MBAS-S total score correlations.

We found a similar pattern of correlations with the MDDI. There were positive moderate to high significant correlations between both questionnaires, with the Worry About Imperfection showing the highest correlations with the MDDI, and the Hope for Perfection factor showing the lowest correlations. As can be seen in Table 3, the Appearance Intolerance and the MDDI total score showed the highest correlations with the PAPS-S. Lastly, there was a positive moderate significant correlation between all PAPS-S scores and CET-S total score.

Finally, Tables 4, 5 show the results of multiple hierarchical analyses. The final regression models for each dependent variable accounted for 24–67% of the variance of PAPS scores. Regarding the general physical appearance perfectionism (PAPS-S total), the independent variables that made a significant contribution were general perfectionism (MPS total); general male body dissatisfaction (MBAS-S total); compulsive exercise (CET-S total); eating, weight, and shape concerns (EDE-Q: EWSC); restraint behaviors (EDE-Q: R); muscle body dissatisfaction (MDDI); and functional impairment (MDDI FI).

**TABLE 4:** Multiple hierarchical regression: overall model effect.

Dependent variable	Final model	*F*	*R* ^2^
PAPS total score	Model 7	139.09***	0.55
PAPS WAI score	Model 5	311.22***	0.67
PAPS HFP score	Model 7	50.38***	0.24

***p < 0.001.

**TABLE 5:** Multiple hierarchical regression: final regression coefficients for each dependent variable.

Dependent variable	Independent variable	Final unstandardized coefficients	Final β (standardized coefficients)	*t*
PAPS total score	Intercept	7.25	–	6.11***
	MBAS total score	1.90	0.16	3.44**
	MPS total score	0.08	0.19	6.91***
	EDE-Q EWSC score	2.77	0.28	7.63***
	MDDI total score	0.42	0.35	6.24***
	EDE-Q R score	–0.75	–0.09	−3.15**
	CET total score	1.02	0.11	3.43**
	MDDI FI score	–0.35	–0.14	−3.11**
PAPS WAI score	Intercept	–1.37	–	12.63***
	MBAS total score	3.04	0.38	8.43***
	MDDI AI score	0.62	0.29	7.46***
	MPS total score	0.05	0.17	−6.84***
	EDE-Q R score	–1.14	–0.22	5.48***
	EDE-Q total score	1.76	0.26	12.63***
PAPS HFP score	Intercept	1.14	–	9.57***
	CET total score	0.04	0.23	6.36***
	MPS total score	0.25	0.16	4.56***
	MDDI DFS score	1.07	0.21	6.19***
	EDE-Q EWSC score	–0.72	0.20	3.70***
	MBAS LBF score	1.14	–0.13	−2.52**

**p < 0.01; ***p < 0.001.

However, as seen in Table 5, there were differences between both PAPS factors (i.e., WAI and HFP) in terms of significant predictive variables, although general perfectionism (MPS) accounted for both factors. On one hand, general male body dissatisfaction; restraint behaviors; general ED attitudes (EDE-Q); and appearance intolerance (MDDI AI) made a significant contribution to WAI factor. On the other hand, compulsive exercise; eating, weight, and shape concerns; drive for size (MDDI-DFS); and low body fat (MBAS LBF) made a significant contribution to HFP factor. It is worth mentioning that the BMI did not make a significant contribution to any of the dependent variables.

## Discussion

Scientific research on perfectionism agrees that the best way to understand this complex construct is through the use of measures that incorporate both its positive and negative aspects (Antony et al., 2004). In this line, the PAPS is a multidimensional assessment instrument, with the novelty of measuring a domain in which many people act with perfectionist tendencies: physical appearance (Yang and Stoeber, 2012).

The aim of the present study was to evaluate the psychometric properties of the PAPS-S in a representative sample of male university students in Spain. The factor structure of the scale, its reliability its convergent and concurrent validity, and the associated predictor variables were examined.

The Spanish version of the PAPS showed a two-factor structure in both the EFA and CFA and in the parallel analysis, confirming the model of the original questionnaire. In our study, item 5 showed cross-loading on both factors, which influences the fit of the RMSEA index. However, removing the item did not significantly improve the model fit, so we opted for a conservative solution, keeping all the original items, and retaining appropriate values of the CFI and TLI fit indices of the factor model. Item 5 also showed fit problems in the Brazilian validation study of PAPS (Neves et al., 2019). However, to not further impoverish the short version of the Brazilian PAPS (i.e., without items 1 and 2) the authors also opted to retain it along with item 8, since its removal did not improve the explained variance of the questionnaire (Neves et al., 2019). Even with the slight modifications of the Brazilian version, the PAPS shows in all translations to date an unambiguous two-factor structure. Moreover, these studies have been conducted in mixed samples in which no differences in questionnaire functioning have been found between males and females, unlike other instruments related to body dissatisfaction that have been used interchangeably among males and females even though the content of the items is clearly biased toward a female body perspective (e.g., Body Shape Questionnaire). Thus, the PAPS has been shown to be a robust instrument in its factor structure and the content of its items has been shown to be apparently neutral in terms of gender differences.

Regarding reliability, the PAPS-S showed high reliability indices in its different translations, with Cronbach’s alpha values above 0.80 in the Chinese, English, and Brazilian version (Yang and Stoeber, 2012; Neves et al., 2019). Although they did not conduct a factor analysis, also a recent study in a mixed sample of adults in the Philippines showed reliability scores above 0.88 on the PAPS (Simon et al., 2022), supporting the reliability of the test. For the Spanish version, the questionnaire showed Omega values of 0.94 for the total scale and the two factors. Although comparisons are limited by the difference in the reliability index calculated in the different studies, the PAPS is confirmed as a highly reliable instrument in all its translations.

In terms of convergent validity, the results of the PAPS-S are related to those of the MPS without being the same construct, confirming the multidimensionality of perfectionism. Our results confirm that the negative dimension of physical appearance-oriented perfectionism (i.e., WAI subscale) is more closely associated with the negative aspects of general perfectionism (i.e., Fear of Mistakes, Parental Influence), in line with previous research (Stoeber and Yang, 2015; Bergunde and Dritschel, 2020). In addition, the positive dimension of the PAPS-S (i.e., HFP subscale) was more associated with the Achievement Expectations and Organization subscales of the MPS. However, in line with previous research (Yang and Stoeber, 2012; Stoeber and Yang, 2015; Bergunde and Dritschel, 2020), the difference between the association of the two subscales of the PAPS with the negative aspects of perfectionism is more pronounced than that of both subscales with the positive aspects of perfectionism. Perfectionism is a complex dimension, and its exploration requires a broad view that allows for a deeper exploration also of its positive dimension (Stoeber, 2018). Although perfectionism may have less harmful aspects, it is dangerously close to psychopathological risk factors, so the study of protective factors that block a possible drift toward pathological hyper self-demand is a key element in the development of a new approach.

Concerning concurrent validity, the results confirm the study hypotheses regarding the association of PAPS-S with body dissatisfaction, ED and DM symptomatology, and compulsive exercise. To our knowledge, this is the first study that has explored the relationship between appearance-oriented perfectionism and body dissatisfaction from a male perspective that included muscle-related aspects. The total PAPS-S, and particularly the WAI subscale, was associated strong and positively with MBAS-S total scores, which measures body dissatisfaction in men. The results regarding the association of the PAPS-S with the MDDI are similar, with higher associations of the WAI subscale of the PAPS-S with the body dissatisfaction subscales of the MDDI. Only the Functional Impairment subscale of the MDDI shows a stronger association with the HFP subscale. This subscale of the MDDI is primarily related to prioritizing the training routine over other activities and the associated impact of not training. Thus, the pursuit of physical perfection may lead people to have a more rigid relationship with their physical activity to the point of impacting on other spheres (e.g., social). On the other hand, although the PAPS-S was associated with ED symptomatology, the low relationship with the Restraint subscale as opposed to the Concern subscales of the EDE-Q, particularly with the HFP subscale of the PAPS-S, was remarkable. The fact that the PAPS-S score is not related to explicit eating disordered behaviors suggests that the PAPS is a measure more oriented to body image concerns in a negative sense beyond the relationship with weight and shape and that, although it may include it, it has not yet translated into clear risk behaviors and associated impairment. Another possible explanation may have to do with the male sample being less prone to restraint behaviors as explored by the EDE-Q (i.e., anorexia nervosa) and more oriented to muscularity-oriented EDs (Murray et al., 2012). In any case, the correlations of the ED and DM questionnaires are higher for the PAPS-S and its subscales than for the general perfectionism measure, supporting data from previous research that suggests that the use of domain-specific measures of appearance-oriented perfectionism explains higher percentages of variance in eating symptomatology and is a better predictor of psychopathology than a generic measure (Stoeber and Yang, 2015; Bergunde and Dritschel, 2020; Czepiel and Koopman, 2021; McComb and Mills, 2021). Finally, the relationship between PAPS-S and CET-S shows fewer differences between the positive and negative dimension of the questionnaire with relation to compulsive exercise. This result is not surprising given that the motives that lead young people to exercise may be varied and related to both health and psychopathological components derived from body dissatisfaction and difficulties in emotional regulation characteristic of EDs and DM (Sicilia et al., 2021). Even so, the findings associated with functional impairment resulting from a rigid training routine place appearance-oriented perfectionism as a relevant study factor in the field of exercise-related pathology.

Regarding the study of predictor variables associated with the PAPS-S, the final model explains 55% of the variance of the total scale, 67% of the WAI subscale and 24% of the HFP subscale. In the literature on perfectionism, perfectionistic concerns are considered more maladaptive and associated with psychopathological variables (Yang et al., 2017; Simon et al., 2022). Since the variables in our study are of this nature, it is not surprising that the variance explained for the WAI subscale across the applied questionnaires is higher than in the case of HFP, which may have adaptive aspects. In all retained regression models, total MPS plays a significant role as a predictor of appearance-oriented perfectionism in general, and of the WAI and HFP subscales. Previous research has used PAPS as a predictor of eating symptomatology (Stoeber and Yang, 2015) and body dissatisfaction (Yang et al., 2017), finding that BMI plays a significant role as a predictor variable. However, to date this is the first study to perform a predictive model of appearance-oriented perfectionism as a dependent variable, with BMI not being a significant predictor. In contrast, for the PAPS total scale, male body dissatisfaction, dissatisfaction with muscularity, weight and shape concerns, restrictive eating behaviors, compulsive exercise and associated functional impairment are relevant predictors. Regarding the WAI subscale, body dissatisfaction, appearance intolerance, food restriction and ED symptomatology act as predictors. On the other hand, compulsive exercise, drive for size, weight and shape concerns, and rejection of body fat and the feeling of fatness influence as predictors in the HFP subscale. This result is interesting, as HFP is apparently associated with perfectionistic strivings that are not necessarily pathological. However, the presence of compulsive exercise and functional impairment derived from a high emphasis on routine exercise is of considerable concern, particularly in the male population who tend to be more physically active and at higher risk of exercise-related pathology (Author et al., 2022).

### Limitations and Future Research

Despites its contributions, this study has several limitations. The present study examined perfectionism as a multidimensional construct based on the Frost et al. (1990) model. Although this model is one of the most widely used in the field of the study of perfectionism, further studies from the perspective of Hewitt and Flett’s (1991) model, or other more recent models (e.g., Hill et al., 2004) may help to extend the research results. In addition, the study was conducted in a sample of university students, which affects the generalizability of the results in other populations (e.g., men with ED or MD) or age groups (e.g., middle aged men, adolescents). Although the validation studies in English and Brazilian Portuguese found no differences between men and women, the results of the present study do not guarantee the reliability of the scale in Spanish women, so it would be desirable to include them in future research. Finally, the study design was cross-sectional and therefore causal relationships between variables cannot be established. Longitudinal studies in this regard are required in the future.

## Conclusion

In conclusion, the PAPS is a valid and reliable instrument for use in a Spanish-speaking male population. Given the multidimensional nature of perfectionism, its use is also recommended using its two subscales. Appearance-oriented perfectionism is associated in males with the presence of body dissatisfaction, risk of ED and MD, and compulsive exercise. These variables are more strongly associated with the WAI subscale. However, risk behaviors such as compulsive exercise act as significant predictors for the HFP subscale. It is hoped that this validation will contribute to improve knowledge about perfectionism oriented to physical appearance, one of its less studied manifestations.

## Data Availability Statement

The raw data supporting the conclusions of this article will be made available by the authors, without undue reservation.

## Ethics Statement

The studies involving human participants were reviewed and approved by the Ethics Committee of the Autonomous University of Madrid (CEI-75-1368). The patients/participants provided their written informed consent to participate in this study.

## Author Contributions

RR: investigation, visualization, project administration, writing – original draft. AM-E: recruitment, formal analysis, results writing. MS: recruitment, formal analysis, results revision original draft. SF: recruitment, writing – review draft. EC: supervision, writing – review and editing. AS: conceptualization, methodology, recruitment, supervision, writing – review and editing. All authors contributed to the article and approved the submitted version.

## Conflict of Interest

The authors declare that the research was conducted in the absence of any commercial or financial relationships that could be construed as a potential conflict of interest.

## Publisher’s Note

All claims expressed in this article are solely those of the authors and do not necessarily represent those of their affiliated organizations, or those of the publisher, the editors and the reviewers. Any product that may be evaluated in this article, or claim that may be made by its manufacturer, is not guaranteed or endorsed by the publisher.

## Annex 1

### Physical Appearance Perfectionism Scale – Spanish version

**ANNEX 1:** Lea cada oración y decida en qué medida está de acuerdo o en desacuerdo. Si está muy de acuerdo debe rodear el 5. Si está muy en desacuerdo, debe rodear el 1. Para una respuesta intermedia debe rodear del 2 al 4. Si se siente neutral al respecto o no está seguro debe rodear el 3, el número central.

	Muy en desacuerdo				Muy de acuerdo
(1) No estoy satisfecho/a con mi apariencia física.	1	2	3	4	5
(2) Espero que la forma de mi cuerpo sea perfecta.	1	2	3	4	5
(3) No importa cómo me vista, nunca estoy contento/a con mi apariencia física.	1	2	3	4	5
(4) Espero ser atractivo/a.	1	2	3	4	5
(5) Me preocupa que mi apariencia física no sea lo suficientemente buena.	1	2	3	4	5
(6) Espero que otras personas admiren mi apariencia física.	1	2	3	4	5
(7) Espero que otras personas me encuentren atractivo/a.	1	2	3	4	5
(8) Desearía poder cambiar por completo mi apariencia física.	1	2	3	4	5
(9) Mi apariencia física está lejos de mis expectativas.	1	2	3	4	5
(10) Me preocupa que otras personas critiquen mi apariencia física.	1	2	3	4	5
(11) A menudo pienso sobre los defectos de mi apariencia física.	1	2	3	4	5
(12) Espero ser guapo/a.	1	2	3	4	5
